# Is Malaria a Water-Borne Disease?

**Published:** 1897-09

**Authors:** John M. Batten

**Affiliations:** Pittsburg, Pa.


### IS MALARIA A WATER-BORNE DISEASE?

BY JOHN M. BATTEN, M. D., PITTSBURG, PA.

Presented to the Section on Practice of Medicine at the Forty-eighth Annual Meeting of the American Medical Association held at Philadelphia, June 1—4, 1897.

Malaria may, to some extent, be a water-borne disease, but that the malaria plasmodium or poison gains admittance into the system wholly by way of the stomach, we think we have ample evidence to lead us to doubt. The plasmodium malariæ or malarial poison certainly gains admittance into the system through the respiratory organs as well.

I will give a hasty review of the disease as it came under my observation in 1864 and 1865. The Valley City, to which I was attached, had a roving permission to cruise in the sounds of North Carolina and up the rivers that empty into these sounds. The water of the sounds at times is brackish, as it has connection with the ocean through Ocracoke Inlet. The intensity of the disease and the number of cases that occurred increased in the following localities in the order named: 1, the region of the Pamlico river, upon which Washington, N. C., is situated, is rather free from malaria, though cases occur there; 2, the region of Chowan river, Blackwater river, Meherrin river, and 3, the regions of Roanoke river and Neuse river. Again, on Roanoke island there is no vegetation except in the northern part of it, where there are a few scrub oaks. The island is sandy and barren. Yet, one day on that island, with Dr. Walton of the 103d Pennsylvania regiment, I saw a hundred cases of malaria, but many of these cases may have been transferred from other localities. The water used there for drinking and cooking purposes was boiled water taken from the wells on the island.

The water for drinking and cooking purposes on the Valley City was either boiled or distilled, taken from the boiler, cooled and kept in a covered vessel for that purpose. Now, when the Valley City was for a long time up the Roanoke river or other rivers, the cases increased in number and intensity according to the locality in which she cruised. When cruising up the Roanoke river for a long time the treatment of the disease in many instances would be of no avail, but afterward, when cruising in the sounds or the Atlantic Ocean the disease would respond to the treatment so that there would not be a single case of malaria on board. Afterward, cruising up any of the aforesaid rivers the number of cases and the intensity would increase according to the locality. In the region of the Roanoke river it was intensely malarious, as forest swamps and fogs prevailed there, and many places in this region were impenetrable to man or even the wild beasts of that locality. Why was there a difference in the type of the disease and also the number attacked while in these different localities? It was not the water we drank nor the food we ate, for the water we drank and used for cooking purposes was boiled or distilled. The boiler received its water by pumping from the river, sounds or ocean. Therefore the malarial poison must have been taken into the system through the respiratory organs.

In the fall of 1865, at Cairo, Ill., following a very hot and dry summer, the months of September and October were exceptionally dry and hot. I reported for duty on the United States monitor Oneota, lying off Cairo on the Ohio river, Oct. 6, 1865. Malarial fever was prevalent everywhere in that vicinity. A large barge moored to the Oneota was filled with cases of malarial fever of a pernicious type. We used water for drinking and cooking purposes that was boiled or distilled as on the Valley City. What I want to show particularly is, not only that malarial fever prevailed notwithstanding that the water for drinking and cooking purposes was boiled, but that it is not necessary to have a wet season follow a dry and warm summer to produce malarial fever or the plasmodium malariæ. Up to Oct. 6, 1865, there had been no rain. The Ohio and Mississippi rivers were very low, and along the banks of the Ohio in the vicinity of Cairo there was a green scum on the surface of the river extending some distance from the shore. Yet, as I have said, there was malarial fever prevailing everywhere in that locality.

Again, in the Western States and in Pennsylvania malarial fever has been actually ploughed out of existence. The agriculturist who, for the first time, turned up the soil suffered the most from malaria, but as the soil was more and more stirred up the less severe and the less frequent would be the attacks, so that to-day in many localities where the disease was most prevalent, there are no cases of malaria. Was it the water that these early settlers drank, the food they ate, or was it the air they breathed by which the plasmodium malariæ or malarial parasites were conveyed into their system.

I hope I have made myself plain on this subject. It is only by observation and experience that we can arrive at a true understanding in regard to this matter. The more plasmodium malariæ that is taken into the system, the oftener the paroxysms of fever will recur, as every day, every second day, etc. Pernicious fever occurs in localities in which the plasmodium malariæ or malarial poison is most intense and most concentrated.—*Journal American Medical Association.*

